# Voxel-based meta-analysis of gray matter and white matter changes in patients with spinocerebellar ataxia type 3

**DOI:** 10.3389/fneur.2023.1197822

**Published:** 2023-07-27

**Authors:** Hai Liu, Junyu Lin, Huifang Shang

**Affiliations:** ^1^Department of Neurology, Laboratory of Neurodegenerative Disorders, Rare Disease Center, West China Hospital, Sichuan University, Chengdu, Sichuan, China; ^2^Department of Neurology, Xuanhan County People's Hospital, Dazhou, Sichuan, China

**Keywords:** voxel-based morphometrics, spinocerebellar ataxia type 3, gray matter, white matter, meta-analysis

## Abstract

**Purpose:**

Increasing neuroimaging studies have revealed gray matter (GM) and white matter (WM) anomalies of several brain regions by voxel-based morphometry (VBM) studies on patients with spinocerebellar ataxia type 3 (SCA3); however, the findings of previous studies on SCA3 patients by VBM studies remain inconsistent. The study aimed to identify consistent findings of gray matter (GM) and white matter (WM) changes in SCA3 patients by voxel-wise meta-analysis of whole-brain VBM studies.

**Methods:**

VBM studies comparing GM or WM changes in SCA3 patients and healthy controls (HCs) were retrieved from PubMed, Embase, Web of Science, and Medline databases from January 1990 to February 2023. Manual searches were also conducted, and authors of studies were contacted for additional data. The coordinates with significant differences in GM and WM between SCA3 patients and HCs were extracted from each cluster. A meta-analysis was performed using anisotropic effect size-based signed differential mapping (AES-SDM) software.

**Results:**

A total of seven studies comprising 160 SCA3 patients and 165 HCs were included in the GM volume meta-analysis. Three studies comprising 57 SCA3 patients and 63 HCs were included for WM volume meta-analysis. Compared with HC subjects, the reduced GM volume in SCA3 patients was found in the bilateral cerebellar hemispheres, cerebellar vermis, pons, right lingual gyrus, and right fusiform gyrus. The decreased WM volume was mainly concentrated in the bilateral cerebellar hemispheres, right corticospinal tract, middle cerebellar peduncles, cerebellar vermis, and left lingual gyrus. No increased density or volume of any brain structures was found. In the jackknife sensitivity analysis, the results remained largely robust.

**Conclusion:**

Our meta-analysis clearly found the shrinkage of GM and WM volume in patients with SCA3. These lesions are involved in ataxia symptoms, abnormal eye movements, visual impairment, cognitive impairment, and affective disorders. The findings can explain the clinical manifestations and provide a morphological basis for SCA3.

## Introduction

Spinocerebellar ataxia (SCA) is a subset of autosomal dominant inherited ataxia which is highly genetic and clinically heterogeneous. Motor coordination disorder is the main manifestation of SCA (1). Other features, such as pyramidal tract signs, extrapyramidal signs, ocular muscle paralysis, cognitive dysfunction, psychiatric symptoms, and dysfunction of touch, vision, hearing, and proprioception, can also occur (1, 2). Currently, more than 48 subtypes of SCA have been identified. SCA Type 3 (SCA3)/Machado–Joseph disease is the most common type (3), which is a trinucleotide (CAG) repeat disease, also known as a polyglutamine disease (4).

The voxel-based morphometry (VBM) technique can identify the differences in the corresponding anatomical structures including the density or volume changes in the gray matter (GM) and white matter (WM) in the brain. It is a whole-brain-based unbiased image post-processing technology and is not affected by different operators. In recent years, many VBM studies have investigated the changes in brain structures between patients with SCA3 and healthy controls (HCs) (5–18). However, the results of these studies were inconsistent or even contradictory. Two studies using VBM demonstrated a volumetric reduction of GM in the cerebellum and brainstem of SCA3 patients (4, 8). Another study found that SCA3 patients had significant GM atrophy in the cerebellar hemispheres and vermis, while no significant WM volume reduction was observed (7). One study found a decreased density of GM in the frontal, parietal, temporal, and occipital lobes, as well as subcortical GM, in addition, decreased GM in the cerebellum and brainstem, and restricted WM volume reduction existed only in the cerebellum (5). However, some studies found that the change in WM volume exists not only in the cerebellum but also in the pons, middle cerebellar peduncles, and other connective structures (6, 8, 14). These inconsistent findings may be because the subjects in each study were uneven or the sample size was relatively small. Recently, a systematic review of 29 studies described GMV changes in the cerebral cortex, subcortical, brainstem, and cerebellar and WMV change in the cerebellar region in SCA3 patients compared to HCs and revealed moderate correlations between MRI features in these regions and clinical manifestations of ataxia, non-ataxia, and cognitive function (19). Eight of these studies analyzed VBM changes in damaged brain areas in patients with SCA3 (5, 8–11, 13, 18, 20). However, this review has several shortcomings. First, different MRI techniques have not been consistently studied across different brain regions in the SCA3 population, specifically the cerebrum. Second, correlations between clinical impairment and brain regions were not consistently reported. Based on the above findings, it is necessary to identify the consistent characteristic imaging changes in the SCA3 subtype.

The anisotropic effect size-based signed differential mapping (AES-SDM) is a voxel-based quantitative meta-analysis method. It can collate and record the MNI peak coordinates of brain regions with statistically significant differences between groups based on whole-brain analysis, can reconstruct the effect scale and statistical parameter map of increased or decreased GM and WM in each original study, and is widely used in various neurodegenerative diseases, such as amyotrophic lateral sclerosis (21), Alzheimer's disease (22), and multiple system atrophy (23), as well as in SCA2 (24). Therefore, the present study aimed to voxel-wisely meta-analyze the GMV and WMV changes in SCA3 patients, respectively, using the ES-SDM method.

## Materials and methods

### Data sources, screening, and data extraction

A comprehensive search of PubMed, Embase, Web of Science, and Medline databases from January 1990 to February 2023 was conducted. Keywords include (“Spinocerebellar Ataxia Type 3” OR “Spinocerebellar Ataxia 3s” OR “Spinocerebellar Ataxia 3” OR “Type 3 Spinocerebellar Ataxia” OR “Machado Joseph Disease” OR “machado-Joseph Disease” OR “Azorean Disease”) AND (“voxel-based morphometry” OR “VBM”). Additional searches were also performed in reference lists.

Inclusion criteria were as follows: (1) Whole-brain VBM results were reported in SCA3 patients compared with HCs in GM volume or WM volume; (2) studies reported the whole-brain results of the changes in three-dimensional coordinates (x, y, and z) in standard stereotactic space; (3) for studies that met the inclusion criteria and had overlapping samples, only the study with the largest sample size was included to avoid duplicate data. Studies were excluded if (1) only a case reports enrollment; (2) there was no HC group; (3) only regions of interest (ROI) were used instead of the whole brain; (4) the necessary information was missing, and the stereotaxic coordinates of the report were not obtained even though we corresponded with the authors by email; and (5) data overlap with data from another article.

Following PRISM procedures and requirements (25), two neurologists (Hai Liu and Junyu Lin) independently searched, screened, and evaluated the literature. In each study, coordinates and their effect sizes (*t*-statistics, *z*-scores, or *p*-values) with significant differences between patients and HCs in GM and WM volume were extracted according to the AES-SDM software tutorial (26). If the results and opinions were inconsistent, they were discussed with each other or arbitrated by a third neurologist (Huifang Shang).

### Voxel-based meta-analysis (VBM)

Previous reports have described the meta-approach in detail (26, 27), and we can also use the SDM package (www.sdmproject.com) to learn the ES-SDM tutorial to compare the differences between GM and WM volume between SCA3 patients and HCs. First, a folder was created containing text files with coordinates and effect sizes for each study. After specifying this folder, an SDM table was created containing the sample size and clinical characteristics of the study. Then, a mean analysis was conducted to compare the GMV and WMV between SCA3 patients and HCs. The default kernel size and statistical thresholds [full width at half maximum (FWHM) = 20 mm, *p* = 0.005, peak height threshold = 1, and extent threshold = 10] were used, which have been validated to optimize the sensitivity and specificity and produce a desirable balance between Type I and II error rates (26, 28). The SDM software editor was also contacted by email when necessary.

A whole-brain voxel-based jackknife sensitivity analysis was conducted to test the replicability of the results. This consists of repeating the main statistical analysis seven times but systematically removing one different study each time to ensure that no single study will bias the combined results and recalculating the stability of the remaining studies. *Q*-statistics were calculated to assess the heterogeneity between studies. Egger's tests were carried out to detect potential publication bias (*p* < 0.05 indicates obvious publication bias), and funnel plots were established for visual inspection.

Meta-regression analyses were carried out to examine the potential confounding variables such as age, disease duration, SARA scores, ICARS scores, and CAG Repeats. Linear model analyses are a generalization of the mean analysis to allow comparisons between groups and the study of possible confounds.

## Results

### Included studies and sample characteristics

**Figure 1:**
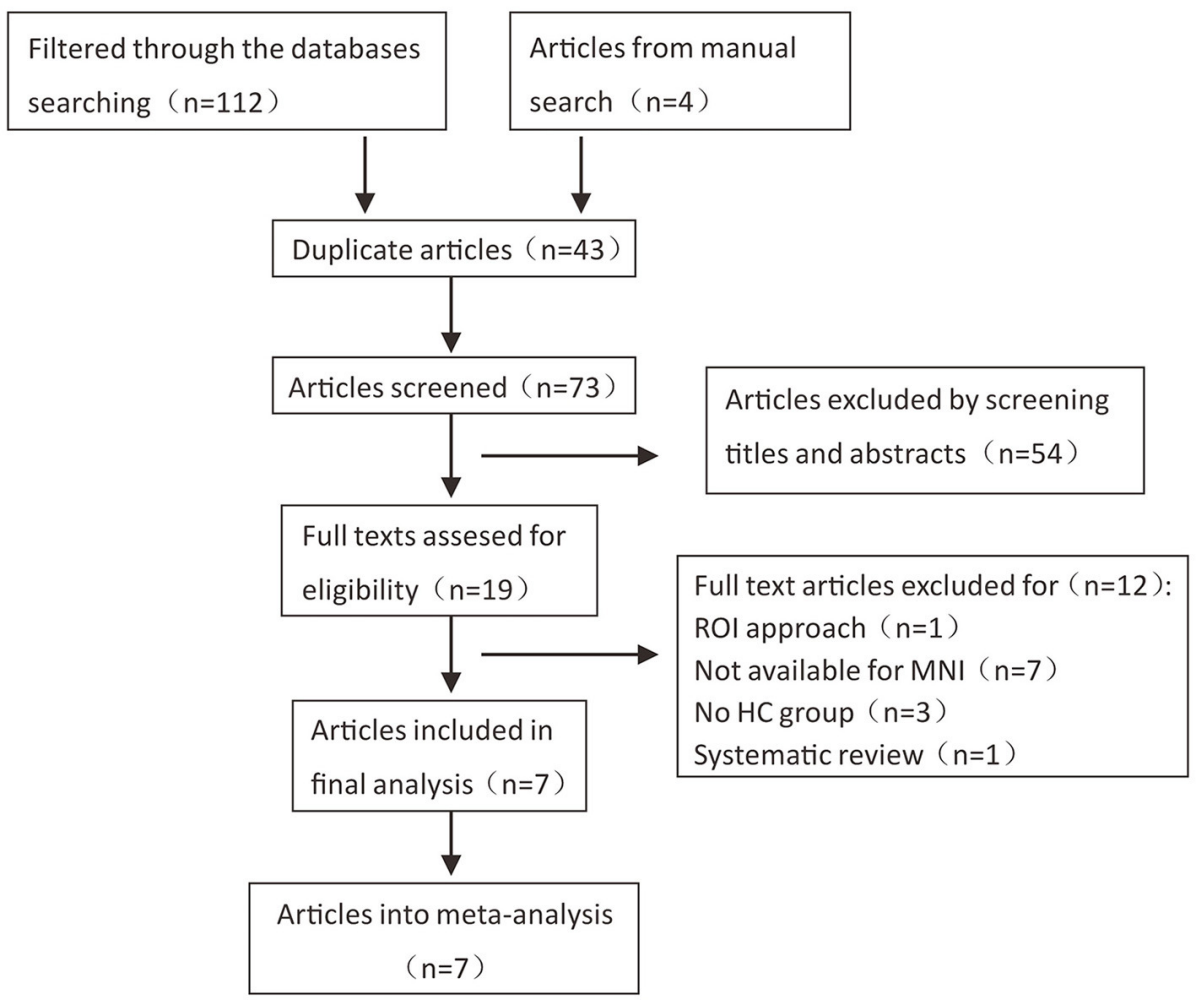
Flow chart of the literature search in the meta-analysis.

According to the literature search strategy, 116 potentially relevant articles were retrieved, and 109 were excluded for varied reasons (the screening flow chart is shown in Figure 1). A total of seven articles were included (5–8, 10, 14, 15) for GM volume changes in comparison with SCA3 patients and HCs, whereas five of the seven studies (5–8, 14) performed the analysis of differences in the GM and WM volume simultaneously. Two studies were excluded due to a lack of WM stereotactic coordinates (5, 6). One study did not observe significant areas of WM atrophy (7). Therefore, only three studies were included in the comparison analysis of WM volume between SCA3 patients and HCs. Finally, a GM volume comparison meta-analysis of seven studies included a total of 160 SA3 patients and 165 HCs. WM volume meta-analysis of three studies included 57 SCA3 patients and 63 HCs. In each included study, there were no significant differences in the sex and age between SCA3 patients and HC groups. The demographic and clinical characteristics of SCA3 patients of included studies are shown in Table 1.

**Table 1:** Characteristics of the studies included in the current meta-analysis.

**References**	**Sample**	**Sex (M/F)**	**Age (year)**	**Duration (year)**	**CAG Repeats**	**ICARS**	**SARA**	**Software**	**Threshold**
Lukas et al. (14)	SCA3 9	4/5	53 ± 9.8	NA	64 (58–73)	30 ± 12.4	NA	SPM2	*p* < 0.001 corrected
	HC 15	9/6	60 ± 10						
Goel et al. (7)	SCA3 10	5/5	38.0 ± 12.6	5.8 ± 4.9	NA	48.6 ± 36.0	NA	SPM2	*p* < 0.05 corrected
	HC 10	5/5	NA						
D'Abreu et al. (5)	SCA3 45	30/15	47.02 ± 12.23	9.97 ± 6.13	72 (65–81)	36.36 ± 18.5	NA	SPM2	*p* < 0.05 corrected
	HC 51	NA	44.08 ± 11.78						
Guimaraes et al. (8)	SCA3 38	21/17	52.76 ± 12.70	9.3 ± 2.7	68.08 ± 4.5	32.08 ± 14.01	14.65 ± 7.33	SPM8	*p* < 0.05 corrected
	HC 38	20/18	48.86 ± 12.07						
Hernandez-Castillo et al. (10)	SCA3 17	7/10	40.1 ± 11.9	6.9 ± 4.4	NA	NA	13.26 ± 7.23	FSL	*p* < 0.05 corrected
	HC 17	7/10	NA						
Meles et al. (15)	SCA3 17	9/8	35.6 ± 10.2	9.7 ± 7	70 (65.5–72.5)	NA	10 (3.5–15.5)	SPM12	*p* < 0.05 corrected
	HC 16	8/8	NA						
Gitai et al. (6)	SCA3 24	12/12	46.1 ± 14.7	8.8 ± 5.2	71.8 ± 4.5 (63–80)	NA	16 ± 6.6	SPSSv15.0	*p* < 0.05 corrected
	HC 18	6/12	45.9 ± 16.6						

### Regional difference in GM and WM volume

**Figure 2:**
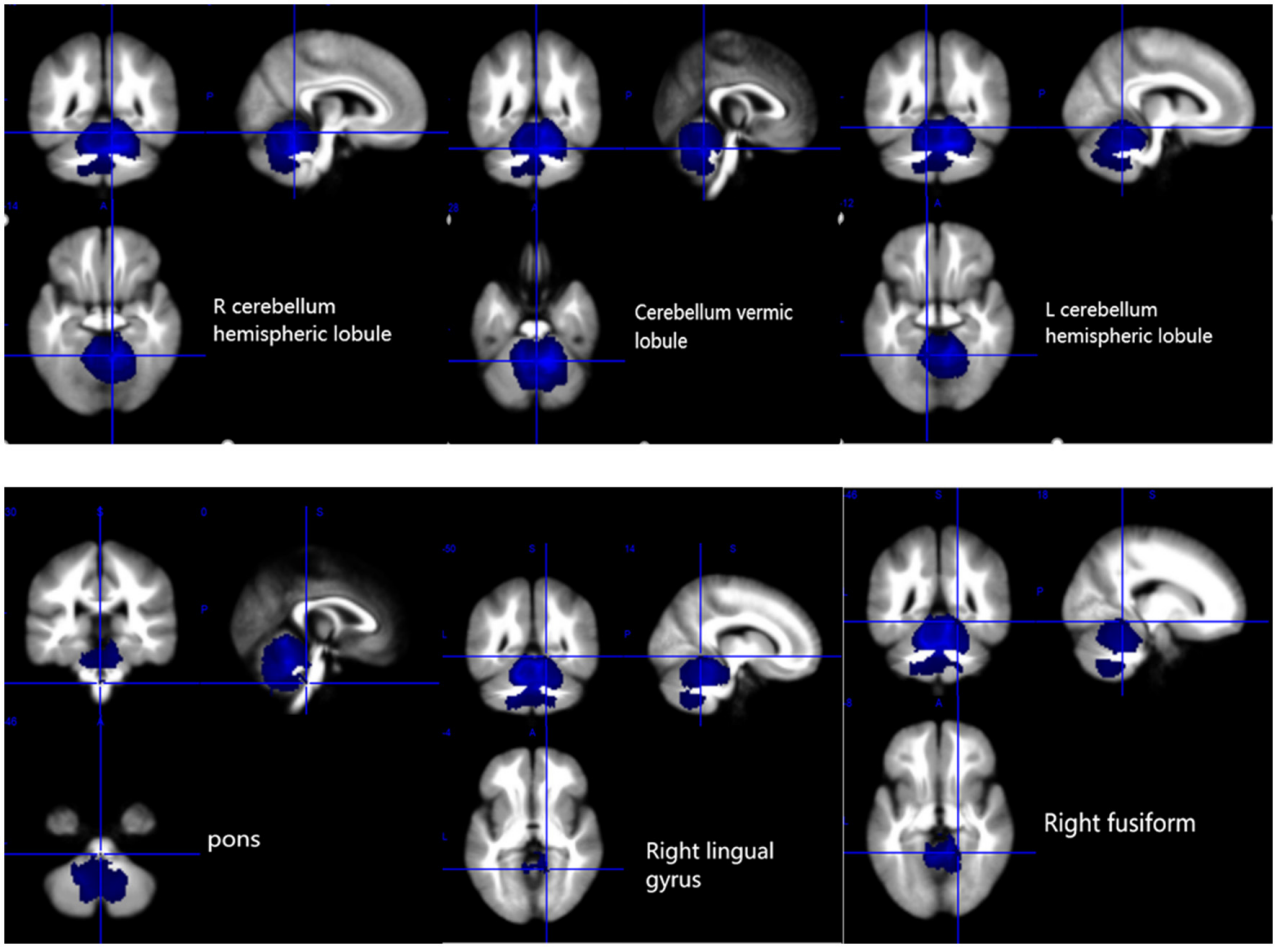
Gray matter atrophy in the bilateral cerebellar hemispheres, cerebellar vermis, pons, right lingual gyrus, and right fusiform gyrus. R, right; L, left.

**Figure 3:**
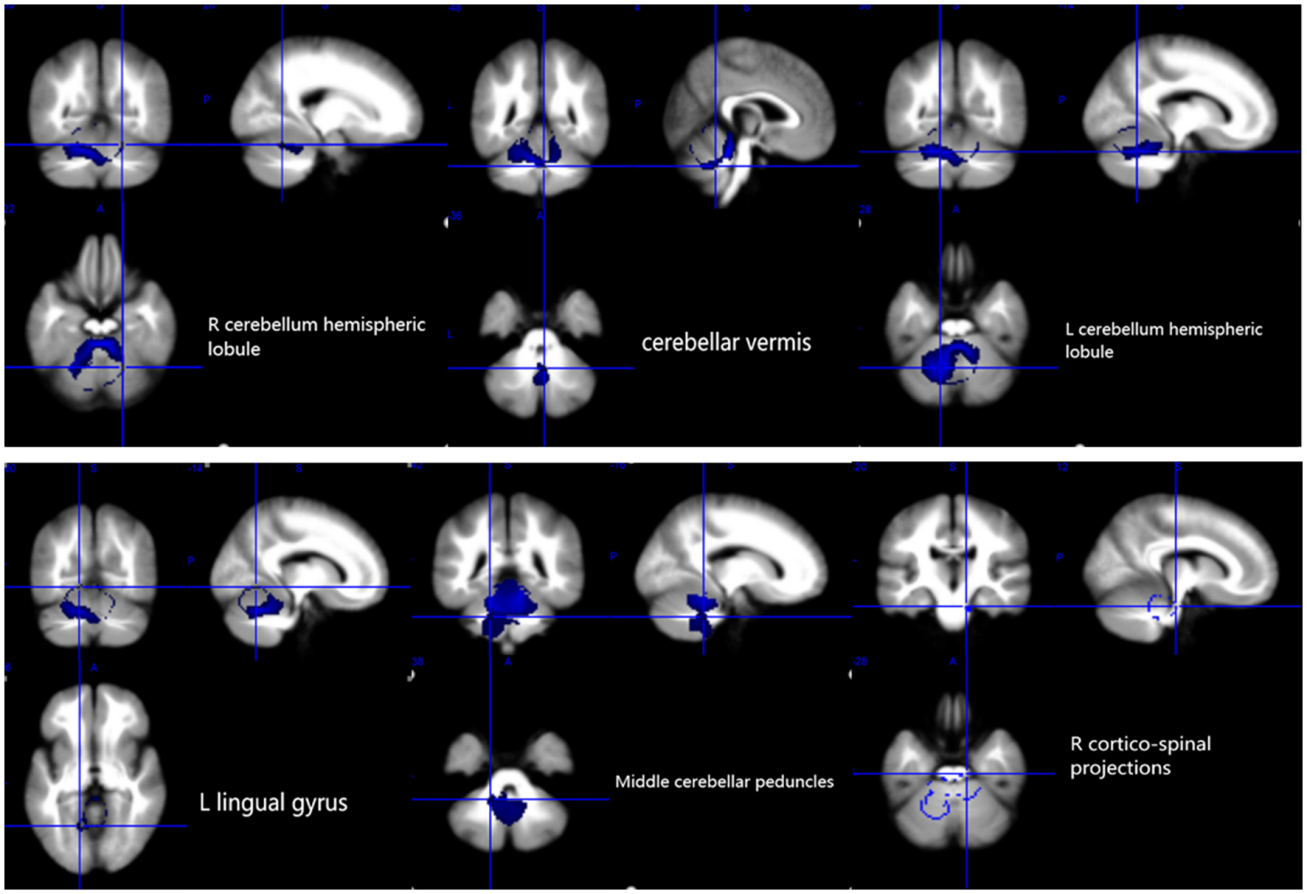
White matter atrophy in the bilateral cerebellar hemispheres, cerebellar vermis, L lingual gyrus, middle cerebellar peduncles, and R corticospinal projections. R, right; L, left.

The results of the voxel-based meta-analysis are shown in Table 2. Compared with HCs, areas of GM volume change in the SCA3 patients are shown in Figure 2: GM volume reduction in the bilateral cerebellar hemispheres, cerebellar vermis, pons, right lingual gyrus, and right fusiform gyrus. Reduced WM volumes were found in the bilateral cerebellar hemispheres, right corticospinal tract, middle cerebellar peduncles, cerebellar vermis, and left lingual gyrus (shown in Figure 3). Compared with HCs, no increased GM or WM volume or density was found in any brain region.

**Table 2:** Regional differences in the GMV and WMV between patients with SCA3 and HCs.

**Regions**	**Maximum MNI coordinates (x, y, and z)**	**No. of voxels**	**SDM-Z value**	***P*-value**	**Egger test (*p*-value)**	**Cluster'breakdown (no. of voxels>50)**	**Jackknife sensitivity**
GM: Cerebellum hemispheric Cerebellum vermic/pons R fusiform gyrus R lingual gyrus	8, −48, −14	8,446	−6.583	~0	0.908	• L cerebellum hemispheric lobule III-VI, VIII/IX R cerebellum hemispheric lobule III-VI, VIII/IX Cerebellum vermic lobule III-VI, VIII/X pons Right fusiform gyrus Right lingual gyrus	• 7 out of 7 7 out of 7 7 out of 7 7 out of 7 7 out of 7 7 out of 7
WM: cerebellum hemispheric R corticospinal projections Middle cerebellar peduncles Cerebellum vermic Left lingual gyrus	−4, −54, −38	726	−1.885	~0	0.790	• L cerebellum hemispheric lobule R corticospinal projections Middle cerebellar peduncles R cerebellum hemispheric lobule Cerebellum vermic lobule IV/V Left lingual gyrus	• 2 out of 3 2 out of 3 2 out of 3 2 out of 3 2 out of 3 2 out of 3

### Sensitivity analyses

As shown in Table 2, these GMV reduction regions were highly reproducible in the jackknife sensitivity analysis because these results were retained in the seven combination studies. For WM analysis, these WMV reduction regions also remained largely unchanged in the jackknife sensitivity analysis.

### Heterogeneity and publication bias

**Figure 4:**
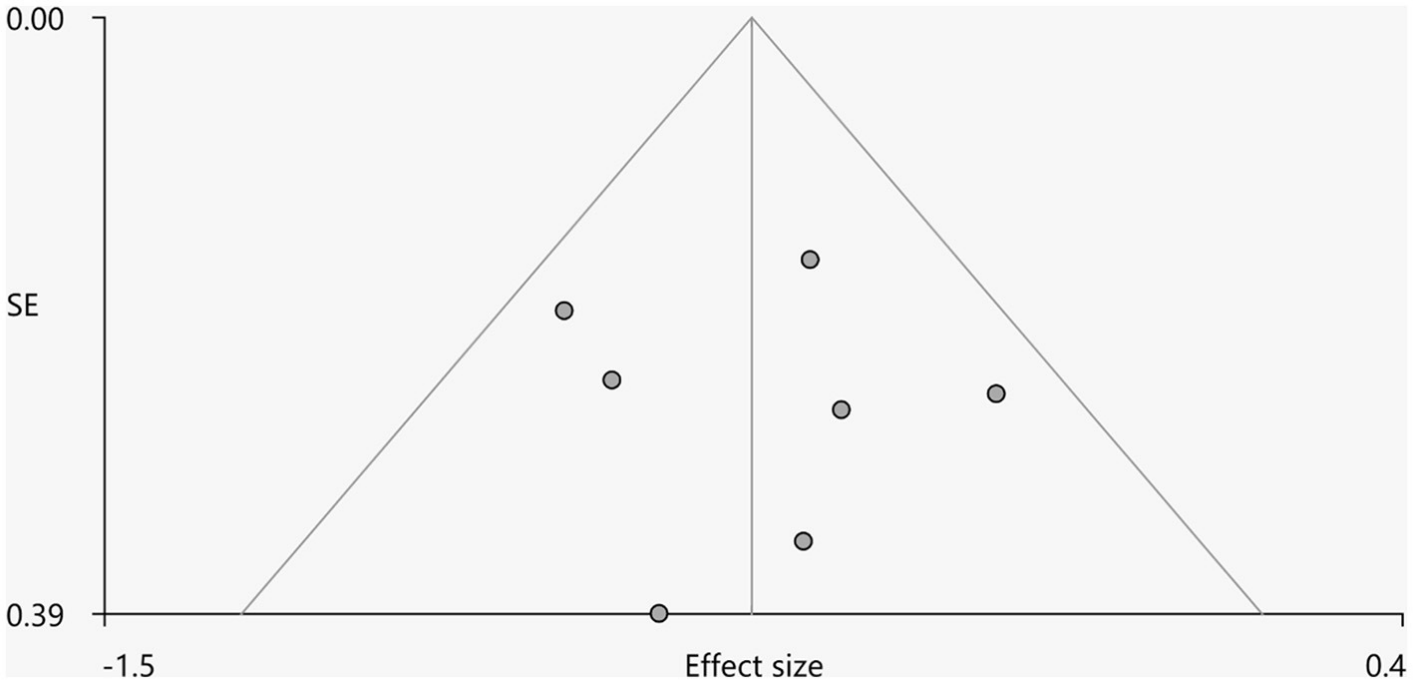
Funnel plot for the peak of the cluster of statistically significantly smaller GMV in cerebellar hemispheres.

**Figure 5:**
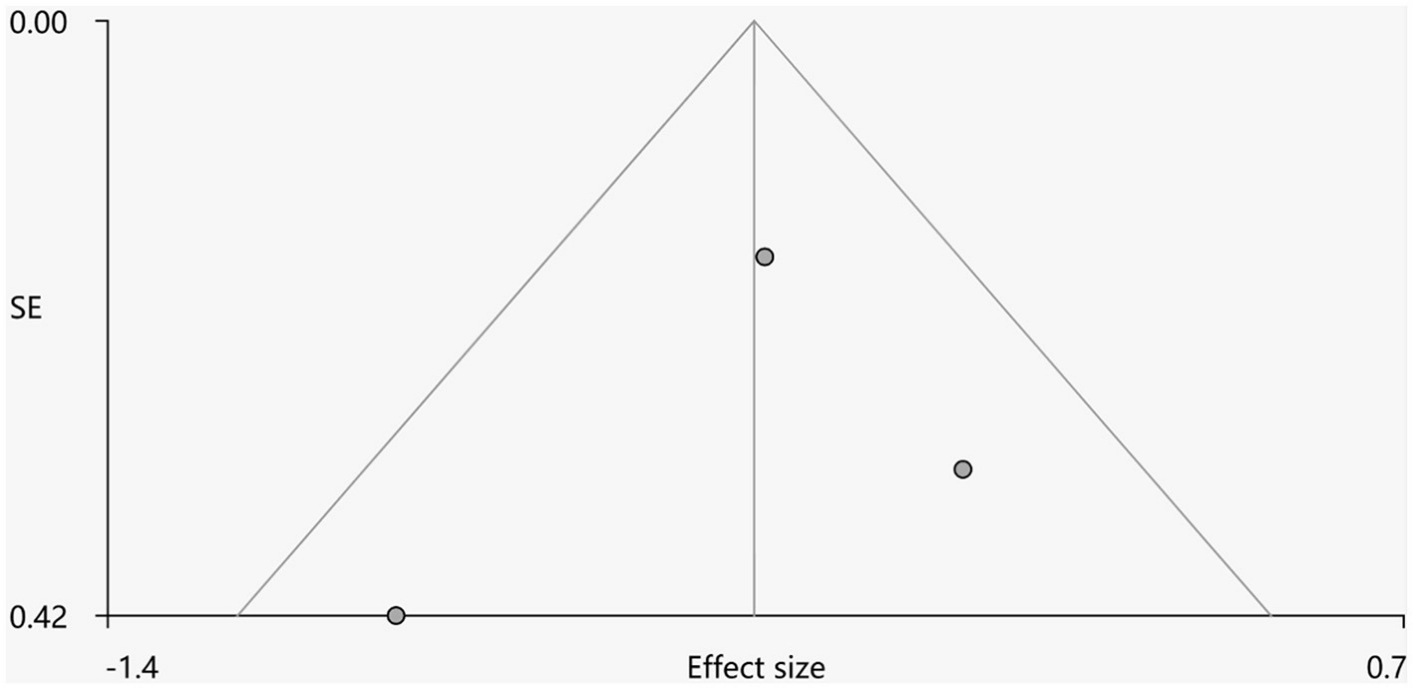
Funnel plot for the peak of the cluster of statistically significantly smaller WMV in cerebellar hemispheres.

Heterogeneity analysis using *Q* statistics indicated that there was no variability between studies. Its exact values, however, should be taken with caution as the recreation of maps from peak coordinates might result in highly inflated statistics. The funnel plot showed that all important brain regions are essentially symmetrical (Figures 4, 5). Egger's test quantitative assessment showed that there was no publication bias in the GMV and WMV of SCA3 (Table 2).

### Meta-regression analyses

Meta-regression analyses showed that older age, prolonged disease duration, and higher SARA scores of SCA3 patients were associated with severe GM atrophy of the cerebellar lobule and lumbrical lobule. Meanwhile, the length of CAG repeats was correlated with the atrophy of the right lingual gyrus and cerebellar vermis lobule in SCA3 patients. The volume of the right lingual gyrus and cerebellar vermis lobule were negatively correlated with the number of CAG repeats. There was a negative correlation between the GMV of the right Rolandic operculum, left insula and right precentral gyrus, and ICARS of patients with SCA3 (Table 3).

**Table 3:** Meta-regression analyses of the potential confounding variables such as age, disease duration, CAG repeats, ICARS, and SARA on GM abnormalities in patients with SCA3.

**Regions**	**Maximum MNI coordinates (x, y, and z)**	**No. of voxels**	**SDM-Z value**	***P*-value**
**Age**				
Cerebellum, vermic lobule IV/V				
Left cerebellum, hemispheric lobule IV/V	−2, −44, −20	830	−5.247	~0
**Disease duration**				
Cerebellum, vermic lobule				
Left cerebellum, hemispheric lobule III	2, −46, −18	82	−1.351	0.002466857
**Cag repeats**				
Right lingual gyrus				
Cerebellum, vermic lobule	4, −32, −8	407	−2.554	0.000005186
**ICARS**				
Right Rolandic operculum	48, −14, 12	746	−2.372	0.001073420
Left insula	−40, −14, 6	204	−2.603	0.000309646
Right precentral gyrus	48, 4, 32	174	−2.641	0.000252903
**SARA**				
Right cerebellum, hemispheric lobule				
Left cerebellum, hemispheric lobule	−2, −40, −20	2,027	−4.096	~0
Cerebellum, vermic lobule				

## Discussion

In this study, voxel-based meta-analysis identified consistent regions of GM and WM volume changes in SCA3. Compared with HCs, GM atrophy of SCA3 patients was mainly detected in the cerebellum, pons, right lingual gyrus, and fusiform gyrus. The reliability of the results was also confirmed by jackknife sensitivity analysis. Reduced WM volumes were found in the cerebellar medulla, cerebellar afferent fibers, right corticospinal tract, and left lingual gyrus.

For infratentorial structures, there is a significant and symmetric GM volume reduction in the cerebellar region, which is consistent with the neuropathological, imaging, and clinical features of patients with SCA3 (19). A previous meta-analysis confirmed the presence of sensorimotor areas (anterior lobes and lobules VIII) and cognitive areas (posterior lobes and particularly lobules VI and VII) in the cerebellum (29). In this study, significant reduction volumes of GM are identified in the cerebellar hemispheric lobule III–VI and cerebellar hemispheric lobule VIII/IX in SCA3 patients. These findings can well explain the clinical manifestations of the patients with SCA3 such as ataxia and cognitive dysfunction. The anterior lobe (lobules I–V) and lobule VIII of the cerebellum are predominantly sensorimotor. The cerebellar motor syndrome results when lesions involve these regions, interrupting cerebellar communication with cerebral and spinal motor systems (29, 30). Cerebellar lobule VI is correlated with visuospatial, verbal memory, and executive tasks (29). Cerebellar lobule IX is considered essential for visual guidance of movement (30, 31), and its damage can cause balance disorder and abnormal gait (10, 32).

In recent years, it has been reported that abnormal static and dynamic eye movements are potential biomarkers of SCA3 (33). Many types of oculomotor adaptation require an intact cerebellum for their normal performance (34), whereas the cerebellar vermis lobule VI/VII is primarily involved in the control of eye movement (35). In an animal study, experimental removal of the cerebellar vermis in monkeys caused impaired binocular motor function and impaired adaptation to esotropia and phoria (36). The results of this study showed that cerebellar vermis cortex atrophy was mainly concentrated in the vermis lobules III-VI and VIII/X, and WM atrophy occurred in lobules IV/V. It has also been reported that the vermis atrophy of SCA3 is more obvious than that of SCA1 and SCA2 patients (7). This may also serve as a basis for early identification of other SCA subtypes.

GM atrophy of the pons is another important finding in the study. Previous studies have noted that atrophy of the pons, particularly the pontine tegmentum, is more pronounced in SCA3 patients than in SCA6 patients (14). Pontine tegmental atrophy occurs early in the clinical onset of the disease and affects the basal part of the pons later. The region-specific changes in this atrophy process may result from differences in the sensitivity of specific cell types and neurons to the pathogenic process (37).

This study also demonstrated damage to the middle cerebellar peduncle and corticospinal tract in SCA3 patients. The middle cerebellar peduncle is an important structure connecting the cerebellum and pons and the main afferent pathway of the cerebellum, also known as the pontine arm, which originates from the WM fiber composition of the contralateral pontine nucleus. Some studies have also confirmed that in diseases such as SCA, preferential loss of Purkinje cells or GM nuclei in the brain stem, resulting in axonal loss and degeneration of related cerebellar middle foot fibers, maybe the mechanism of neurodegeneration affecting MCP (38). Damage or disruption of these structures connecting the cerebellum and the cerebral motor cortex in patients with SCA3 similarly results in severe ataxia and other symptoms (39). It has also been mentioned earlier that patients with SCA3 are accompanied by pyramidal tract signs, and the corticospinal tract is the largest descending motor pathway in the pyramidal tract. Studies using 3.0T diffusion tensor imaging (DTI) have observed damage to the corticospinal tract in patients with SCA3, and the microstructural damage may be related to the severity of ataxia in the disease (40). This study also confirmed the involvement of the corticospinal tract in SCA3 patients, consistent with the findings of Bodranghien et al., that the cause of severe ataxia is not only cerebellar damage (41).

A previous study showed that SCA3 patients suffered damage not only to infratentorial structures but also to supratentorial brain regions such as the frontal lobe, parietal lobe, and temporal lobe (5). This conclusion is corroborated by the GM atrophy in the lingual and fusiform gyrus observed in this study. Anatomically, the lingual gyrus belongs to the ventral striate region and is the center of visual recognition, episodic memory, and emotional control (42). The visual recognition network includes the cuneus, lingual gyrus, fusiform gyrus, and lateral occipital cortex (43). The fusiform and lingual gyri have been reported to encode visuospatial information (44). The fusiform gyrus is mainly related to the visual processing neural pathways and is also closely related to memory, cognition, and emotion perception (45, 46). This also explains the extracerebellar symptoms seen in SCA3 patients. Studies have shown that cerebellum and brainstem damage may precede the brain in patients with SCA3, with neuropathological changes gradually affecting the basal ganglia and cerebral cortex as the disease progresses (19, 47). This process also aggravates the cognitive impairment of SCA3 patients (48). This study only found GM volume reduction in the fusiform gyrus and lingual gyrus but not in the frontal and parietal lobes, which may be related to the small sample size included and the short disease duration of the subjects in enrolled studies. In addition, none of the included patients with SCA3 were followed up until death for autopsy. Unfortunately, due to the lack of necessary relevant information, we excluded some studies that might have been included, such as partial studies that lacked stereotaxic coordinates (6, 18). Although these studies were not included, their findings are still worthy of our attention. For example, one study found significant changes in the cerebellum and the brainstem GM volume between SCA3 patients and healthy controls (6). Another study showed a reduction in gray matter volume in the cerebellum hemispheres and vermis, but little reduction in the pons, basal ganglia, and cerebral hemispheres. The decrease in white matter volume mainly occurred in the brainstem, pons, midbrain, cerebellar peduncles, and cerebellar hemispheres (18). These results are somewhat different from the results of our study. In patients with SCA3, cerebellar lesions may be more damaged than other brain regions and gradually affect brain structure as the disease progresses. Based on this, more longitudinal studies are expected to provide evidence for the development process of the disease in the future, and meta-analysis based on a large number of original studies will be more reliable.

## Limitations

There are several limitations to the current study. First, there was no more comprehensive inclusion of studies other than those published in English, excluding studies for which we were unable to extract stereotaxical coordinates. The small number of included articles limited the scope of this study, which may have led to bias. Second, the included SCA3 patients had a short disease duration range and were mostly cross-sectional studies, which may have missed some areas of volume atrophy during disease progression. In addition, due to the small sample size of the studies included in the literature, no subgroup analysis was conducted.

## Conclusion

The results of the meta-analysis showed that compared with HC, the reduction of GMV in SCA3 patients was not limited to the cerebellum but also mainly involved the lingual gyrus and fusiform gyrus. However, WM atrophy was mainly found in the cerebellum and its afferent pathways and pyramidal tracts. These findings have important implications and may help identify SCA3 from other subtypes of spinocerebellar ataxia. It is helpful for early detection, diagnosis, and intervention and provides a morphological basis for further studies on SCA3.

## Data availability statement

The original contributions presented in the study are included in the article/supplementary material, further inquiries can be directed to the corresponding author.

## Author contributions

HL: concept and design, data collection, data analysis, and drafting of the manuscript. JL: data collection, data analysis, and critical review of the manuscript. HS: authenticity of all the raw data, revision of the manuscript and critique, and overall content as the guarantor. All authors contributed to the article and approved the submitted version.
